# Shaping the Future of Dental Education: A Scoping Review of Artificial Intelligence (AI) Integration Strategies

**DOI:** 10.7759/cureus.84921

**Published:** 2025-05-27

**Authors:** Fawziah Ahmed A Alzahrani, Lubna Alolaiwi, Sulaiman A Alshammari

**Affiliations:** 1 Department of Dental Public Health, College of Medicine and Dentistry, Riyadh Elm University, Riyadh, SAU; 2 Department of Dentistry, Riyadh Elm University, Riyadh, SAU; 3 Department of Family and Community Medicine, College of Medicine, King Saud University, Riyadh, SAU

**Keywords:** artificial intelligence, curriculum integration, dental education, diagnostic tools, educator readiness, ethical challenges, personalized learning, regulatory frameworks

## Abstract

Dental education is one of the fields that have been impacted by artificial intelligence (AI), with new trends such as adaptive learning, virtual simulation, and better diagnostic tools. Despite the many opportunities to use AI in curricula and clinical settings, AI integration has its limitations, highlighted by concerns and challenges such as ethical issues, current and future legal frameworks, and heterogeneity in the preparedness of educators and learners. This study presents a scoping critique of the AI initiatives in dental education and training, as well as analyses of its possibilities, effects, issues, and trends that may inform practice- and research-based curricular improvement and AI preparedness. The current research is based on data obtained from 15 studies included in the scoping review, comprising systematic reviews, observational studies, cross-sectional surveys, and literature reviews, all published in English between January 2020 and January 2024. Basic information was garnered on the features of the studies, the AI technologies and applications, the objects and samples, the results, and the limitations. Thematic synthesis identified four primary areas of focus: curriculum integration, diagnosing and clinical objectives, educators' and students’ preparedness, and ethical and legislation concerns. The review also emphasizes how AI can revolutionize dental education by making learning individualized, diagnosis better, and providing new instruments in teaching. Specific conclusions focus on the recent curriculum necessity in schools, increased preparedness of educators and students, and ethical considerations of bias and data protection. However, there is still very low awareness and training among the employees, which does not support readiness. Ethical and regulatory issues are still among the most important obstacles. This paper therefore argues that only when these gaps are addressed can AI be fully integrated and begin to revolutionize dental education and practice. AI is believed to have great benefits for dentistry education and practice. To fully realize its potential, it must offer educators and students attention to some of the gaps they may not be fully prepared for, devise a set of ethical rules, and establish effective regulatory norms. The results suggest that large-scale, multicenter studies must be conducted to replicate results and explore the full potential of AI integration. This review goes beyond listing AI tools by critically assessing their effectiveness in curriculum design, diagnostic accuracy, and institutional readiness. It synthesizes thematic findings to guide future implementation and policy frameworks in dental education.

## Introduction and background

AI evolution is progressing at a high rate and is becoming the new era in industries, and dental education is going to take a huge leap in the near future [1]. Through the advancement in artificial intelligence (AI), dentistry stands to benefit greatly from increased effectiveness in the way content, knowledge, and skills may be imparted, learned, or even applied. From adaptive learning solutions that let learners locate information according to their unique needs to advanced diagnostic tools that can enrich and enhance the dental education process by closing the gap between learning theory and practice [2]. Such innovations are revolutionizing the conventional perspectives of education with the preparations of future dentists to work in the advanced technological and economical health care context.

As for technologies, virtual simulations, learning platforms, and assessment tools, that use an AI component, are already presenting a change in medical and dental training [3]. Such tools allow the learners to perform elaborate processes virtually and safely, letting them get ready for the actual clinical practice [3]. In addition, AI supports personalized learning environments while fitting the educational content to each learner’s requirements and enhancing the acquisition of higher-order thinking skills [4]. This integration not only trains the student for clinical practice effectiveness but also exposes them to the use of technology in clinical practice especially AI in dentistry.

There are certain challenges in the process of AI incorporation within dental education. Privacy, fairness concerns, and biases are still challenging questions that need to be solved to make an efficient application of AI possible [5]. Furthermore, there are no common practice and research-based guidelines that would help educators integrate AI into their practices without encountering some challenges [5]. In addition, AI integration effectiveness is highly dependent on creating a culture within universities; especially among the faculty and first-year, students in accepting the new transforming tools [5].

This paper aims at reviewing the implementation of AI in dental education critically inspecting the approach that is offered by these technologies as well as the disparities. Based on existing data and regarding the outcomes of various activities used in present training models, this study aims to contribute insights into ‘what works’ in the use of AI for the transformation of dental training. The idea is to provide aspiring dental professionals not only with the best clinical knowledge but also the skills to use advanced AI solutions in their profession to bring value to the patient’s life.

Much of the current literature remains fragmented, with limited comprehensive synthesis of implementation strategies, challenges, and regulatory implications. Therefore, conducting a scoping review that consolidates existing knowledge, evaluates practical trends, and proposes a structured framework for AI integration in dental education is both timely and necessary. This study addresses that gap by offering a detailed thematic analysis aligned with Preferred Reporting Items for Systematic Reviews and Meta-Analyses (PRISMA) guidelines and drawing actionable insights for academic and clinical stakeholders [6].

In the course of this review, the use of an evidence-based approach, considerations of the ethical aspect, and, accessibility to an affordable solution are also highlighted, as well as the need for the sustainable adoption of AI in dental education. Therefore, through designing a future integrated with AI in dental training, this study will promote the generation of a technologically skilled and patient-oriented dental workforce. Therefore, this review evaluates how AI is actively transforming dental education through its impact on diagnosis, individualized learning, and educator/student preparedness. It aims to identify not only current practices but also gaps in implementation and effectiveness.

## Review

Aims of the study

The present scoping review aimed to investigate current AI integration strategies in dental education. Specifically, it sought to evaluate their effectiveness and limitations, identify existing research gaps, explore associated ethical and regulatory challenges, and propose a framework to enhance teaching methodologies and learning outcomes using AI.

Methods

Research Question

The research question guiding this scoping review was: "In dental education (P), how is artificial intelligence (AI) being integrated (I), compared to traditional educational methods or no integration (C), in terms of effectiveness, preparedness, and ethical implications (O)?" (Table 1).

**Table 1:** PICOS criteria AI: artificial intelligence; PICOS: population, intervention, comparison, outcomes, study type

Element	Description
Population (P)	Dental students and educators in academic institutions
Intervention (I)	Use of AI tools (e.g., virtual simulation, machine learning, chatbots) in education or diagnostics
Comparison (C)	Traditional teaching methods or absence of AI integration
Outcomes (O)	Improvement in educational outcomes, diagnostic skills, readiness, and ethical compliance
Study type (S)	Systematic reviews, cross-sectional studies, observational studies, literature reviews

Study Design and Selection Criteria

A scoping review was done to establish a broad understanding of AI implementation in dentistry for education curricula, the AI readiness of educators and students, and the clinical use and implications of using AI in dentistry. In accordance with the PRISMA checklist, this review presented all the necessary information transparently and became reproducible [6]. Only publications from the period of 2020-2024 were included, and articles had to focus on the use of AI in dental education, in diagnoses, or ethical matters.

Data Sources and Search Strategy

A broad literature search was conducted across multiple electronic databases, including PubMed, Scopus, Web of Science, and the Cochrane Library, using keywords such as “artificial intelligence,” “dental education,” “integrated curriculum,” “diagnostics,” and “ethical issues.” Boolean operators (AND, OR) were used to refine the search strategy and maximize coverage. In addition, the reference lists of included articles were manually screened to identify any relevant studies not captured through database searches. The specific search syntax, Boolean combinations, and the number of results retrieved from each database are summarized in Table 2.

**Table 2:** Search strategy and results across databases (2020–2024) AI: artificial intelligence

Database	Search syntax/keywords	Boolean operators used	Number of results retrieved
PubMed	("artificial intelligence" OR "AI") AND ("dental education" OR "dentistry curriculum") AND ("ethics" OR "readiness")	AND, OR	112
Scopus	("artificial intelligence" AND "dental education") OR ("AI" AND "dental training")	AND, OR	135
Web of Science	("AI in dentistry" OR "artificial intelligence in dental education") AND ("integration" OR "curriculum")	AND, OR	98
Cochrane Library	("artificial intelligence" AND "dental education") OR ("diagnostic AI in dentistry")	AND, OR	34
Manual search	Screening of references in key included articles	N/A	8
Total after deduplication	N/A	N/A	15 (included studies)

Inclusion and Exclusion Criteria

Papers selected for this review presented research on AI in dental education, considering topics like integration into curriculum or teaching, preparedness, clinical use, or potential issues regarding AI. This included quantitative and qualitative research papers, systematic reviews, and observational research, which were published in the English language between 2020 and 2024.

On the other hand, works that did not concern specifically dental education or AI, or were commentaries, editorials, or conference abstracts, and those that did not meet essential measures of quality and contain enough data, were excluded. These measures included clarity in study objectives, transparent methodology, relevance to AI in dental education, and the presence of measurable outcomes. “Not enough data” refers to studies lacking details such as the type of AI used, the mode of implementation, participant characteristics, or clearly reported educational or clinical outcomes.

Although studies with moderate to high risk of bias were included when they contributed valuable thematic insights, studies with critically incomplete reporting, missing empirical findings, or poor methodological transparency were excluded. No strict numerical cutoff was used; instead, the appraisal was based on qualitative assessment using the Joanna Briggs Institute (JBI) critical appraisal and Cochrane risk-of-bias tools to determine overall relevance and rigor [7,8].

Data Extraction

**Figure 1:**
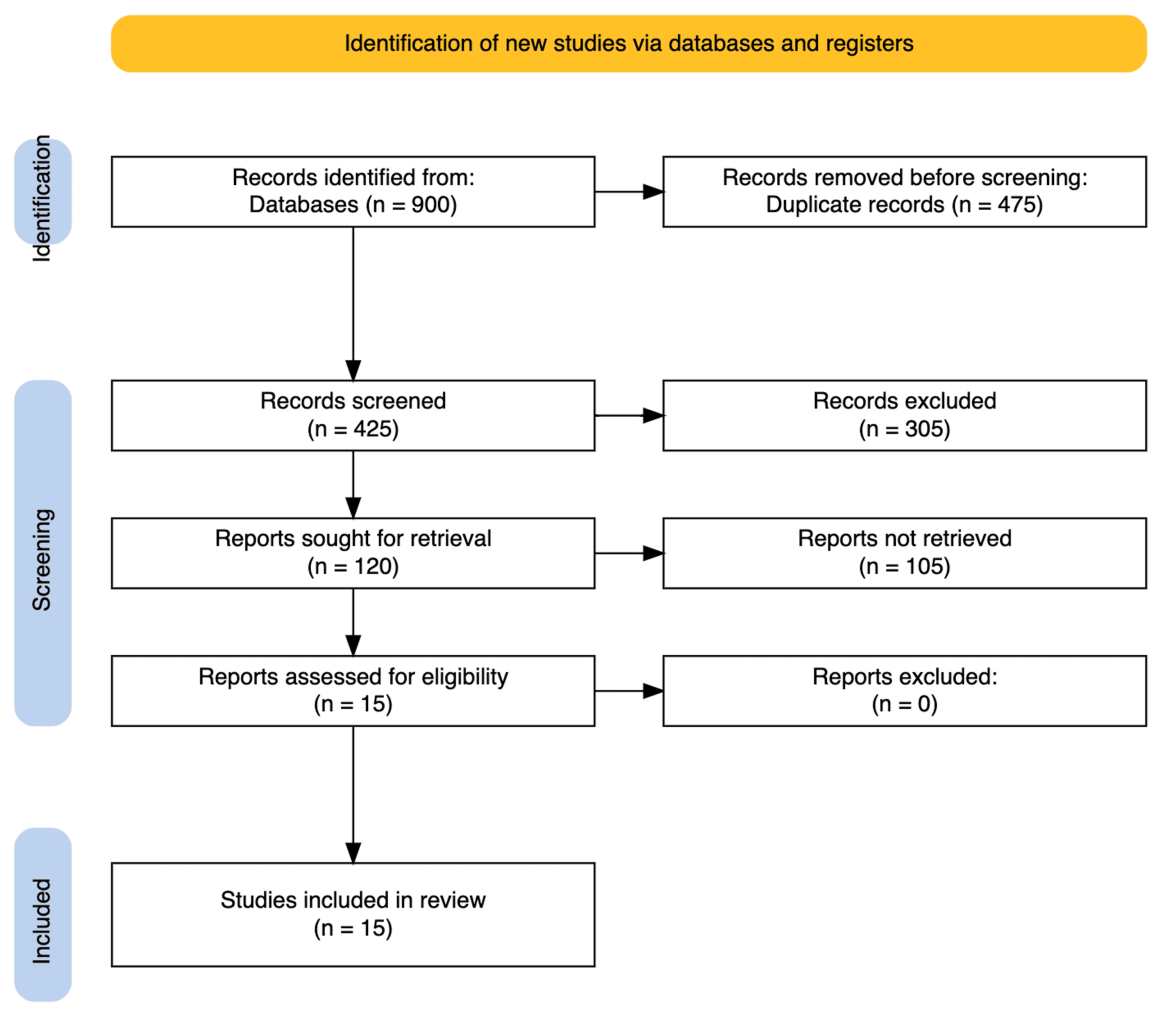
PRISMA flow chart PRISMA: Preferred Reporting Items for Systematic Reviews and Meta-Analyses

Data were extracted in a systematic manner employing a data extraction form that contained several important fields. This included the study reference where the author(s) and the year when the particular study was published and the study design which focused on the method used in the particular study. Further, the form elicited data regarding the country or context of the study to understand geographical and institutional variation. Information about the particular AI technology used in the study is presented along with related articles that discuss the use of technology in dental education. It was observed that the population or participants in each of the studies were pointed out to comprehend the research concerns and coverage or sample type. Last, there was a part of the form compiling the important points about the conclusions and the ideas coming from the study limitations. Figure 1 summarizes the study selection process using the PRISMA flow diagram.

Risk-of-Bias Assessment

Each study was evaluated for risk of bias independently by two reviewers by using the JBI and Cochrane tools [7,8]. These instruments were applied to assess the quality of systematic reviews, observational studies, and cross-sectional designs. Based on this assessment, studies were categorized into low, moderate, or high risk of bias. This systematic evaluation helped to reduce variability and ensure the credibility of included studies.

The possibility of bias in the included studies was generally moderate to high, largely due to the limited availability of high-quality empirical data, methodological inconsistencies, and small sample sizes. Several studies relied on self-reported data, were conducted at single institutions, or lacked clear details on AI interventions, contributing to an increased risk of bias. Additionally, a notable proportion of the included studies originated from Asia and the Middle East. While these regions provided valuable perspectives on AI adoption in dental education, the geographic concentration poses a limitation to the generalizability of findings. Sociopolitical factors, resource availability, institutional digital maturity, and educational infrastructure vary significantly between regions. For instance, North America and Western Europe often operate within more robust regulatory and technological environments, which can influence how AI tools are developed, evaluated, and integrated into curricula. Consequently, the findings of this review may not fully capture experiences in higher-resource settings or offer a balanced global perspective. Broader geographic representation and cross-cultural analyses are needed in future studies to enhance the universality and applicability of insights on AI integration in dental education.

Data Synthesis and Analysis

Data were integrated, analyzed, and interpreted qualitatively based on the identified themes: applications of AI in dental settings; the influence of the applications on dental education; and the implementation experiences captured during the study. Studies were organized into three thematic categories: (1) curriculum and educational integration encompassing educator and student preparedness, (2) diagnostic and clinical use, and (3) ethical and practical issues. While presenting descriptive findings for each thematic area the strengths and weaknesses of the study were also pointed out. Where appropriate, data were summarized numerically to ensure a better focus and also to give a holistic view of the broad facets of AI in dental education.

Risk-of-Bias Summary

**Figure 2:**
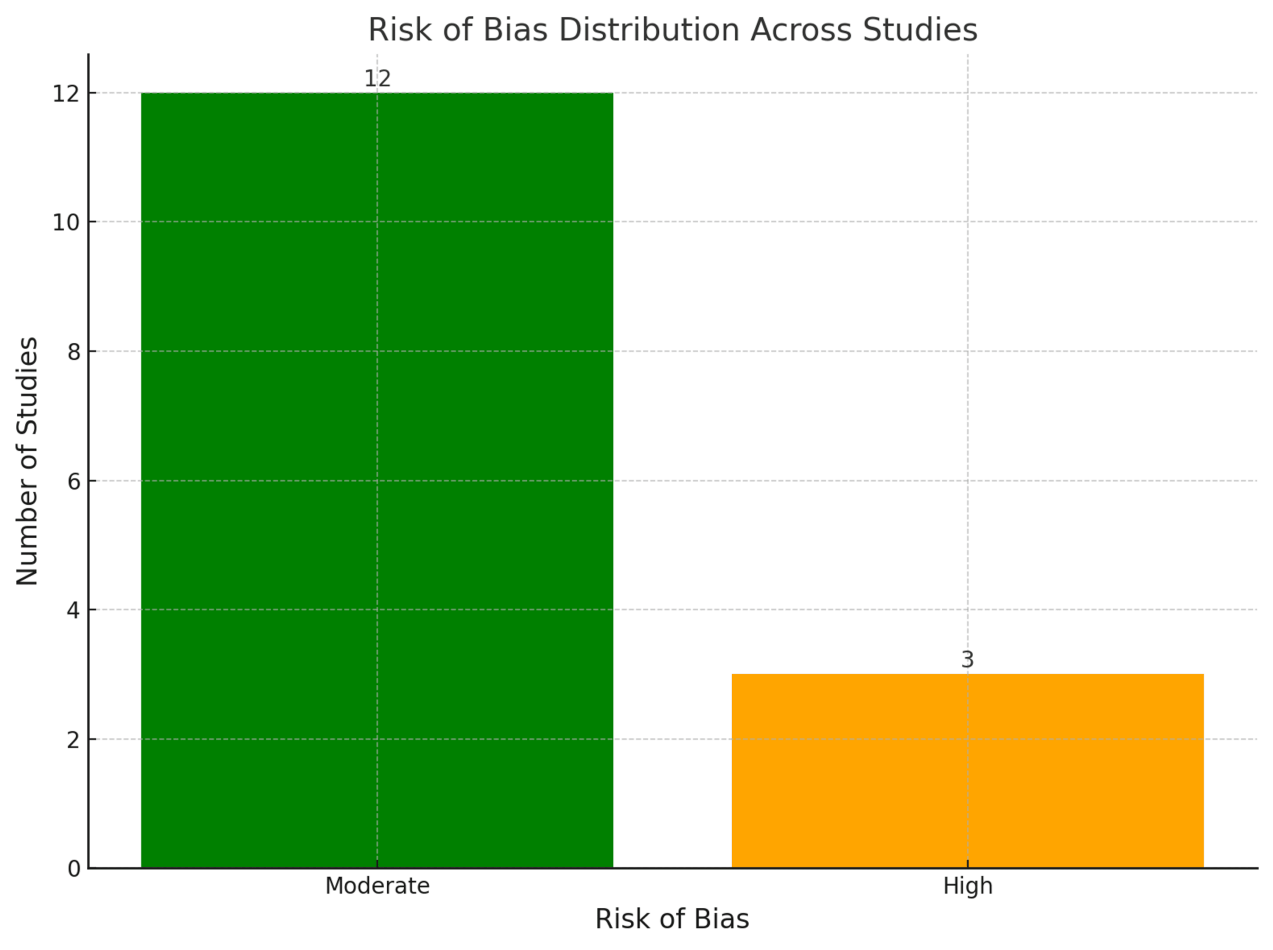
Risk-of-bias distribution across studies The possibility of bias in the included and compared studies was moderate to high owing to the scarcity and nature of the empirical data, geographical generalisability, and methodological quality of the studies. It would be ideal for future studies to avoid these limitations and employ large-sample research with consistent designs and a diverse patient population.

A summary of sources of bias identified in the studies was prepared in a summary table for easy comparison of included studies in terms of their quality. Table 3 summarizes the bias assessment for all included studies and Figure 2 illustrates the distribution of risk of bias across included studies.

**Table 3:** Risk-of-bias assessment across included studies JBI: Joanna Briggs Institute, RCT: randomized control trial, AI: artificial intelligence

Study reference	Study design	Risk-of-bias tool	Domains assessed	Risk of bias (low/moderate/high)	Justification
Saghiri et al. (2022) [9]	Systematic review	JBI checklist for systematic reviews	Study selection, quality assessment, evidence synthesis, reporting transparency	Moderate	Over-reliance on scoping methodologies; limited empirical evidence; insufficient exploration of emerging AI tools.
Thurzo et al. (2023) [10]	Literature review	N/A	Coverage of AI technologies, evidence synthesis	High	Conceptual guidance without practical validation; potential selection and reporting bias due to limited data.
Kim et al. (2023) [11]	Literature review	N/A	Conceptual clarity, evidence synthesis, and practical applicability	High	Theoretical focus without empirical validation; lacks practical examples; potential reporting bias.
Zitzmann et al. (2020) [12]	Systematic review	JBI checklist for systematic reviews	Methodological rigor, study inclusion, and reporting transparency	Moderate	Data largely pre-2020; limited focus on newer AI technologies; variability in study quality.
Carrillo-Perez et al. (2022) [13]	Systematic review	JBI checklist for systematic reviews	Study selection, evidence synthesis, and transparency of reporting	Moderate	Focus on esthetic dentistry; lacks generalizability to broader dental education; variability in study designs.
Khanagar et al. (2021) [14]	Systematic review	JBI checklist for systematic reviews	Methodological rigor, reporting of included studies, evidence synthesis	Moderate	Limited high-quality studies included; variability in methodologies; lack of clinical trials.
Ma et al. (2022) [15]	Literature review	N/A	Clarity of reporting, theoretical framework, and applicability	Moderate	Limited application studies; reliance on theoretical perspectives raises questions of generalizability.
Al-Zubaidi et al. (2024) [16]	Non-RCT observational Study	Cochrane risk-of-bias tool for observational studies	Participant selection, data completeness, reporting of results	Moderate	Small sample size, geographic limitations, and reliance on qualitative data increase the potential bias.
Hammoudi Halat et al. (2024) [17]	Non-RCT observational study	Cochrane risk-of-bias tool for observational studies	Participant selection, reporting of outcomes, and data transparency	High	Small sample size, single institution, and limited demographic diversity increase risk of selection bias.
Kumari M. (2023) [18]	Cross-sectional survey	JBI checklist for cross-sectional studies	Sampling methods, response rate, and generalizability	Moderate	Limited awareness (48.6%); reliance on self-reported data increases response bias; limited generalizability.
Pandey and Malik (2024) [19]	Systematic review	JBI checklist for systematic reviews	Study selection, evidence synthesis, and transparency	Moderate	Ethical and regulatory hurdles persist; limited empirical data supporting broader applications of AI in dentistry.
Dhopte and Bagde (2023) [20]	Review	N/A	Ethical considerations, reporting transparency, practical insights	Moderate	Ethical and regulatory challenges unresolved; limited practical guidance provided for real-world applications.
Ahmed et al. (2021) [21]	Systematic review	JBI checklist for systematic reviews	Study inclusion, evidence synthesis, methodological transparency	Moderate	Lack of clinical validation; insufficient focus on real-world applications; potential over-reliance on theoretical models.
Ra and Kanagaraj (2023) [22]	Literature review	N/A	Clarity of insights, practical applicability, methodological transparency	Moderate	Lack of clinical trials; focus on theoretical models without adequate validation.
Bernauer et al. (2021) [23]	Systematic review	JBI checklist for systematic reviews	Study selection, evidence synthesis, reporting clarity	Moderate	Small sample size, early-stage research; limited focus on long-term outcomes in prosthodontics.

Results

PRISMA Overview

The study followed the PRISMA 2020 guidelines to ensure a transparent and reproducible review process. A total of 387 articles were retrieved from four electronic databases (PubMed, Scopus, Web of Science, and Cochrane Library), with an additional 8 studies identified through handsearching. After removing duplicates and screening titles and abstracts, 120 full-text articles were assessed for eligibility. Of these, 105 were excluded based on predefined inclusion and exclusion criteria. Ultimately, 15 studies were included in the final synthesis. The complete selection process is illustrated in the PRISMA flow diagram (Figure 1), and the reasons for the exclusion of full-text articles are detailed in Table 4.

**Table 4:** Characteristics of included studies RCT: randomized controlled trial, AI: artificial intelligence, AR: augmented reality, VR: virtual reality, ML: machine learning, DL: deep learning, CBCT: cone beam computed tomography

Study reference	Study design	Country/setting	AI technology used	Application in dental education	Population/participants	Outcomes assessed	Key findings	Limitations
Saghiri et al. (2022) [9]	Systematic review	USA	AI and virtual teaching models	Scoping review of AI and immersive technologies	31 studies analyzed	Current applications, potential for integration	AI has vast potential in education; guidelines and methodological improvements are needed for better integration.	Limited studies reviewed; lack of empirical evidence.
Thurzo et al. (2023) [10]	Literature review	Europe	Generative AI, deep learning	Curriculum updates for AI-assisted diagnostics	N/A	Curriculum impact, AI-assisted diagnostics	Curriculum updates are inevitable due to AI growth; ethical concerns persist.	Conceptual guidance without practical validation.
Kim et al. (2023) [11]	Literature review	USA	AI in dental curriculum	Ethical integration of AI into dental education	Conceptual study	Curriculum updates, ethical AI use	Ethical integration of AI is essential for equipping graduates with advanced decision-making skills.	No empirical data; theoretical focus.
Zitzmann et al. (2020) [12]	Systematic review	Global	Digital tools (AR/VR, E-learning)	Digitalization of dental curriculum	82 studies reviewed	Digital tools for knowledge transfer	AR and VR will dominate future dental education, revolutionizing training methods.	Data mostlypre-2020; limited focus on AI.
Carrillo-Perez et al. (2021) [13]	Systematic review	Global	Machine learning (ML), deep learning (DL)	AI applications in diagnostics and esthetic dentistry	120 studies reviewed	Diagnostic systems, esthetics	AI enhances diagnostics and esthetics; excellent decision-support systems for dental restorations.	Focuses on esthetics; limited educational insights.
Khanagar et al. (2021) [14]	Systematic review	Global	Various AI technologies	Advances in diagnostics, imaging, and planning	Studies reviewed	Diagnostic capabilities	Enhanced imaging and diagnostic capabilities through AI in dental sciences.	Limited evidence from high-quality studies.
Ma et al. (2022) [15]	Literature review	Global	Explainable AI	Standardization of AI tools in dentistry	Studies on caries prediction	Standardization, trust in AI	Explainable AI increases diagnostic trust; essential for broader adoption of dental AI solutions.	Limited real-world application studies.
Al-Zubaidi et al. (2024) [16]	Non-RCT observational study	Pakistan	AI readiness among faculty	Examining faculty perceptions of AI readiness	21 faculty members	Perceptions, training needs	Faculty readiness varies; AI is seen as beneficial but raises ethical and training concerns.	Small sample size; geographically limited study.
Hammoudi Halat et al. (2024) [17]	Non-RCT observational study	Qatar	AI in healthcare	AI readiness and perceptions in health education	94 dental students	Readiness for AI, educational needs	Students show average readiness; a significant need for AI knowledge and training exists.	Small sample size; single institution.
Kumari M. (2023) [18]	Cross-sectional survey	India	AI educational tools	Integration of AI in dental education	128 dental students	Perception of AI in education	Students have a positive perception; enthusiasm for AI tools like virtual simulations is evident.	Limited awareness (48.6%); survey-based limitations.
Dhopte and Bagde (2023) [20]	Review	Global	AI diagnostic tools	Ethical concerns and practical applications	N/A	Potential benefits, ethical challenges	AI holds transformative potential but requires addressing bias, privacy, and regulatory concerns.	Ethical and regulatory challenges unresolved.
Ahmed et al. (2021) [21]	Systematic review	Global	Neural networks, ML	Diagnostics, patient management	32 included articles	Diagnostic precision and efficiency	AI enhances diagnostic precision and efficiency, showing significant potential.	Need for clinical validation and transparency.
Ra and Kanagaraj (2023) [22]	Literature review	Global	Radiographs, CBCT, imaging	Disease detection and educational aids	Research papers analyzed	Disease detection, educational tools	AI tools offer significant benefits for disease detection and learning aids in dental education.	Lack of clinical trials.
Bernauer et al. (2021) [23]	Systematic review	Global	AI for diagnostics, prediction	Prosthodontics and patient-centered treatment	7 studies	Diagnostic tools, predictive measures	Promising AI applications in prosthodontics for diagnostics and prediction.	Small sample size; early-stage research.
Uribe et al. (2024) [24]	Non-RCT observational study	Global	AI chatbots (e.g., ChatGPT)	Perceptions of AI chatbots in dental education	428 dental educators	Educator perceptions	Positive outlook on AI chatbots but concerns about reduced human interaction and lack of guidelines.	Self-reported data; regional variability.

General Characteristics of Included Studies

The final 15 included studies comprised a mix of systematic reviews (6), literature reviews (5), cross-sectional surveys (2), and observational studies (2). The majority were published between 2021 and 2024. Most studies originated from Asia (India, Pakistan, Qatar), the Middle East (Saudi Arabia), Europe, and a few from the United States. A detailed breakdown of study types, AI technologies used, and geographical settings is presented in Table 4.

The AI technologies covered included machine learning (ML), deep learning (DL), virtual simulations, chatbots (e.g., ChatGPT), and adaptive learning tools. Participants were mainly dental students and educators, with sample sizes ranging from 21 to 428 individuals.

Outcome Characteristics of Included Studies

Based on thematic synthesis, the 15 studies were grouped into four major categories, which are detailed below.

Curriculum integration (6 studies): These studies explored how AI is being embedded into dental curricula, highlighting tools like AR/VR, deep learning, and diagnostic models. Common themes included the need for updated syllabi, faculty training, and policy support for AI integration. Authors such as Thurzo et al. and Kim et al. emphasized the urgency of preparing educators and institutions for this transformation [10,11].

Diagnostic and clinical applications (4 studies): Studies in this category demonstrated how AI supports early disease detection, radiographic interpretation, and treatment planning. AI-based imaging tools and explainable AI models were noted for improving clinical decision-making. However, limitations included the lack of clinical trials and validation data.

Educator and student preparedness (3 studies): Research in this group assessed awareness, training needs, and attitudes toward AI among dental students and faculty. While there was broad enthusiasm for AI tools (e.g., virtual simulations, AI chatbots), many respondents reported low preparedness and a lack of institutional training opportunities.

Ethical and regulatory issues (2 studies): These studies identified concerns about bias in algorithms, data privacy, and the absence of comprehensive regulatory standards. Authors such as Dhopte and Bagde and Ahmed et al. called for transparent AI guidelines, approval frameworks, and the inclusion of ethics education within dental curricula [20,21].

A complete distribution of articles across these themes is provided in Table 5.

**Table 5:** Distribution of included studies by thematic category

Thematic category	Number of studies	Studies included
Curriculum integration	6	Saghiri et al. (2022) [9], Thurzo et al. (2023) [10], Kim et al. (2023) [11], Zitzmann et al. (2020) [12], Ma (2022) [15], Kumari M. (2023) [18]
Diagnostic and clinical applications	4	Carrillo-Perez et al. (2022) [13], Khanagar et al. (2021) [14], Ahmed et al. (2021) [21], Bernauer et al. (2021) [23]
Educator and student preparedness	3	Al-Zubaidi et al. (2024) [16], Hammoudi Halat et al. (2024) [17], Uribe et al. (2024) [24]
Ethical and regulatory issues	2	Dhopte and Bagde (2023) [20], Ra and Kanagaraj (2023) [22]

Effectiveness of AI Tools in Key Domains

Diagnosis: Studies by Carrillo-Perez et al. and Khanagar et al. showed that AI-based diagnostic tools improved early disease detection and treatment planning, especially via imaging and deep learning [13,14].

Curriculum delivery:Saghiri et al. and Zitzmann et al. highlighted how AI-supported simulations and adaptive platforms enhanced student engagement and retention [9,12].

Preparedness: Al-Zubaidi et al. and Kumari M. reported increased enthusiasm among faculty and students, but low institutional readiness and lack of training remain key barriers [16,18].

Ethical and legal impact: AI use raises concerns about algorithmic bias and data misuse. Authors like Dhopte and Bagde emphasize the urgent need for ethical frameworks and formal regulation [20].

Discussion

AI in dentistry has become a trend that has created a new way of teaching and practicing dentistry due to the innovations it brought into the teaching and practicing arena. In the 15 included studies, the following thematic findings provide an understanding of the present and future status and possibilities of AI in dentistry education and clinical application.

Curriculum Integration and Future Preparedness

One more constant observed in the analyzed articles is the necessity to include AI technologies in the curricula of dental education. The introduction of generative AI, augmented reality/virtual reality (AR/VR) tools, and deep learning algorithms present a massive opportunity to revolutionize the approach to teaching and clinical training. The works of Saghiri et al. and Thurzo et al. point out the fact that curriculum changes are more than necessary due to the development of AI technology [9,10]. But at the same time, these works raise the need for rational criteria, ethical standards, and effective methods for integration. In addition, as Kim et al. state, ethical issues should be prioritized to better prepare future dentists for managing biases and ensuring accountable, responsible AI implementation in clinical practice [11]. Finally, Zitzmann et al. discussed literature related to the use of technology like AR and VR in learning [12]. Although these technologies present a future vision of radical educational approaches, the overall absence of AI-tailored focus, along with the reliance on data derived before 2020, limits a product’s relevance to present-day AI advancements.

Diagnostic and Clinical Applications

AI technologies have been very instrumental in enhancing diagnostic and clinical decision-making of dental disorders. From enhancing the accuracy of imaging methods to serving as tools for a tailor-made approach to treatment, applications of machine learning (ML) and deep learning (DL) algorithms shine brightly in terms of patient outcomes. Carrillo-Perez et al. and Khanagar et al. are examples of research that expounds AI application in aesthetic dentistry and diagnostic imaging as a new course of treatment [13,14]. Moreover, as has been stressed by Ma et al. explainable AI makes systems more transparent, trust diagnosing AI tools more, and contributes to better adoption of AI by clinicians [15]. These developments support the enhanced patient-centered care resulting from the use of AI and offer insights into the future of technologically aided dental practices.

Educator and Student Readiness

The teacher and learner preparedness factor significantly determines the extent to which human-implicit AI technologies are adopted in dental training. The perceptions observing the faculty are also optimistic about AI, as shown by Al-Zubaidi et al. [16]. However, this article identifies considerable deficiencies in training and support. The study by Hammoudi Halat et al. and Kumari M. also found that students are enthusiastic about using AI tools including the virtual simulations, although they lack awareness of the technology [17,18]. In order to overcome these challenges, extensive, simple, and easily available training sessions, along with organizational approaches toward the implementation of technological solutions must be implemented [18].

Ethical and Regulatory Challenges

Hurdles such as ethical and regulatory limitations are still factors that will slow the full implementation of AI practice or learning in dental applications. It is seen that data privacy, algorithmic bias, and absence of regulation were among the concerns highlighted in most of the studies. For instance, Dhopte and Bagde, urge for the development of approval frameworks that feed transparency, and accountability as well as those that seek to harmonize AI from exclusion [20]. In the same way, Ma et al. and Ahmed et al. also support clinical verification and standardization measures to improve the credibility as well as the fairness of AI solutions [15,21]. Overcoming these ethical and regulatory issues is necessary to restore the confidence in dentistry faculty, practitioners, and patients; these issues are also vital to adapting AI technologies wisely.

Ra and Kanagaraj investigated AI tools as both diagnostic instruments and teaching resources, stressed the advantages, and discussed open issues such as regulatory and ethical status [22]. Finally, Bernauer et al. described several areas of AI implementation in prosthodontics, as well as predictive diagnostics [23]. However, a problem with small sample sizes and the fact that the majority of works discussed in the literature review are still in the first stage of exploring the phenomena under consideration. These were considered major drawbacks, thus underlining the importance of the development of stringent guidelines that would ensure proper consideration of ethical and regulatory issues.

Lastly, Uribe et al. developed positive attitudes toward AI chatbots among 428 dental educators across the world [24]. There was also caution, though, over immediacy and a perceived lack of technical norms and risks associated with less human contact.

These observations taken together once again give an idea about the possibilities that AI holds in dentistry, from reformulating education and training to overhauling clinical applications. But they raise also system-related questions like the call for frameworks of ethical decision-making, the need for training faculty and students, and the development of sound integration models. Overcoming these barriers shall prove pivotal to optimizing the benefits afforded by AI technologies and avoiding any discrimination in the application or implementation of AI in dental education and practice.

Limitations of the Research 

Several limitations were identified in the reviewed studies that may hinder the applicability and generalizability of the findings. The inclusion of diverse study designs, such as systematic reviews, literature reviews, observational studies, and cross-sectional surveys allowed for a broader perspective but also reduced methodological consistency and comparability across studies. In particular, the inclusion of review articles, such as those by Carrillo-Perez et al., which lacked empirical data or measurable outcomes, limited the overall strength of evidence and underscored the need for more clinical trials and longitudinal research designs [13].

Moreover, although a formal risk of bias assessment was conducted using the JBI and Cochrane tools, the results of this appraisal were not quantitatively incorporated into the interpretation of findings. Consequently, studies with moderate to high risk of bias were discussed alongside lower-risk studies, which may affect the robustness of thematic conclusions.

In terms of generalizability, studies such as those by Al-Zubaidi et al. and Hammoudi Halat et al. involved small sample sizes and were geographically limited to specific regions [16,17]. These limitations restrict the applicability of findings across broader international contexts. Additionally, differences in AI readiness, educational infrastructure, and cultural perceptions between regions highlight that findings from one context may not be transferable to others [22].

Lastly, although the search strategy included handsearching of reference lists, only eight additional records were identified, and none met the full inclusion criteria. This process was documented to ensure transparency, but no eligible articles were ultimately added through handsearching.

Relevant literature has been cited to support and contextualize these limitations, aligning them with commonly reported challenges in AI integration across dental education research. Overcoming these constraints through rigorous, multicenter studies with diverse populations and consistent methodologies will be essential for advancing evidence-based AI integration in dental education and training.

## Conclusions

This scoping review highlights the untapped potential of adopting AI technologies in dental education and clinical practice. Emerging tools such as generative models, augmented reality, and machine learning offer breakthroughs in diagnostic precision, patient-centered care, and modern teaching methods. Integrating these innovations requires thoughtful curriculum updates, faculty training, and student preparation to build optimism, competence, and ethical awareness.

Despite these promising developments, existing studies are limited by small sample sizes, regional concentration, and predominantly theoretical designs, which reduces generalizability. To advance evidence-based AI integration, there is a clear need for robust multicenter trials, longitudinal research, and the establishment of standardized programs and guidelines. Addressing ethical, regulatory, and practical challenges will be pivotal for successfully embedding AI in dental education and maximizing its long-term impact.
